# Predicting postpartum female sexual interest/arousal disorder via adiponectin and biopsychosocial factors: a cohort-based decision tree study

**DOI:** 10.1038/s41598-025-12025-3

**Published:** 2025-07-27

**Authors:** Saiedeh Sadat Hajimirzaie, Najmeh Tehranian, Amin Golabpour, Ahmad Khosravi, Seyed Abbas Mousavi, Afsaneh Keramat, Mehdi Mirzaii

**Affiliations:** 1 School of Nursing and Midwifery, Shahroud University of Medical Sciences, Shahroud, Iran; 2 School of Nursing and Midwifery, Tehran University of Medical Sciences, Tehran, Iran; 3 Department of Reproductive Health and Midwifery, Faculty of Medical Sciences, Tarbiat Modares University, Tehran, Iran; 4 School of Allied Medical Sciences, Shahroud University of Medical Sciences, Shahroud, Iran; 5 Ophthalmic Epidemiology Research Center, Department of Epidemiology, Shahroud University of Medical Sciences, Shahroud, Iran; 6 Department of Psychiatry, Psychiatry and Behavioral Sciences Research Center, Addiction Institute, Mazandaran University of Medical Sciences, Sari, Iran; 7 Center for Health Related Social and Behavioral Sciences Research, Shahroud University of Medical Sciences, Shahroud, Iran; 8 School of Medicine, Shahroud University of Medical Sciences, Shahroud, Iran

**Keywords:** Sexual dysfunctions, Pregnant women, Postpartum period, Adiponectin, Socioeconomic factors, C4.5 decision tree algorithm, Hormones, Peptides, Psychology

## Abstract

After childbirth, women experience significant psychological, physiological, and hormonal changes. To better diagnose individuals at risk of postpartum complications, predictive models utilizing data mining and machine learning techniques can be instrumental. The C4.5 decision tree algorithm effectively analyzes multiple variables to identify key relationships. The objective of the study was to predict Female Sexual Interest/Arousal Disorder (FSIAD) six months postpartum using serum adiponectin levels and biopsychosocial factors through decision tree analysis. A longitudinal cohort study was conducted with data from 170 pregnant women, collecting data at three points: the third trimester, 40 days postpartum, and six months postpartum. Blood samples were analyzed for adiponectin, estradiol, and testosterone. At the same time, participants completed assessments using the Female Sexual Function Index (FSFI), the World Health Organization Well-Being Index, a socioeconomic index, and a questionnaire on non-biological factors affecting sexual desire. The prevalence of FSIAD was found to be 29.7%, and the model achieved 93.7% accuracy in predicting FSIAD. Significant predictors included serum adiponectin (T1), estrogen (T3), waist circumference (T2, T3), orgasm disorder, and pain disorder, all with p-values < 0.05. The model provides a clinically valuable tool for early identification of at-risk women, allowing for timely intervention and personalized postpartum care.

## Introduction

In the first year postpartum, the prevalence of sexual disorders is estimated at 22–86%, with most women experiencing at least one issue, such as decreased libido, reduced frequency of sexual activity, vaginal dryness, difficulty reaching orgasm, dyspareunia, and sexual dissatisfaction^1,2^. According to previous studies, the most commonly reported disorder is related to sexual desire^3,4^. Furthermore, the Diagnostic and Statistical Manual of Mental Disorders, Fifth Edition (DSM-5) refers to Female Sexual Interest/Arousal Disorder (FSIAD), which is defined as “the lack of, or significantly reduced sexual interest/arousal"^5^. The prevalence of sexual disorders in women, particularly hypoactive sexual desire disorder (HSDD), is high and is influenced by complex interactions of biological, psychological, and social factors, which can be understood through a biopsychosocial model^6^. Estrogen is consistently identified in the literature as a major contributor to female sexual interest^7^. Some evidence suggests a potential link between decreased testosterone levels and female libido, however, there is ambiguous evidence for this hypothesis^4^. An interrelationship has been observed between sex hormones (especially E2 and testosterone) and adipokines in different studies^8^. Compared with other adipokines, adiponectin is secreted mainly by adipose tissue and has a greater density in the blood^9^. The biochemical effect of this peptide partly depends on higher serum levels^10–12^. Earlier findings show, several psychological factors are associated with decreased sexual desire, such as sexual abuse and emotional inattention during childhood, traumatic experiences during puberty, body image, perceived stress, anxiety and aggression, and expectations (prediction) of a negative experience, depression, anorexia nervosa, etc^13^. The social component of the model emphasizes the effect of culture, religious, environmental, and family factors, and the effects of these factors on the incidence of the disease^7,13^. Although the perception regarding the impact of vaginal birth on sexual disorders is one of the most important reasons for women’s desire to have a cesarean section, studies have shown controversial results in this regard^1^. In some studies, the type of delivery has not been found to influence the sexual function of women^14,15^but in others, the mode of delivery significantly impacted sensory dysfunction; cesarean sections showed a neuroprotective effect, while vaginal delivery led to temporary sensory disturbances that resolved within six months postpartum^16^.

To address the numerous biopsychosocial variables associated with sexual dysfunction, a decision tree model was used, known for its effectiveness in classification and prediction. Unlike other predictive models, the decision tree relies on clear (if-then) rules, here is a set of questions in the decision tree, and as each question is specified, another question is asked. If the questions are asked correctly, a short series of questions is enough to predict a new record category. The C4.5 algorithms is a key method for constructing decision trees for classification^17^.

Sexual disorders are important causes of family disintegration, divorce, and infidelity, which can have profound and long-lasting effects on married life^18^. This study is important as it addresses the high prevalence of sexual disorders postpartum, which can significantly impact women’s quality of life and relationship, few studies have investigated the effects of biopsychosocial factors, especially adiponectin hormone on female sexual function in the postpartum period. For this purpose, a decision tree approach was used to predict FSIAD six months postpartum based on serum adiponectin levels and biopsychosocial factors.

## Methods

### Study design and protocol

In a longitudinal cohort study, 170 mothers in their third trimester were selected based on inclusion criteria from those who visited Shahroud Health Care Centers between 2018 and 2019. The sample size of 144 was estimated based on a previous study by Heiman et al., which compared hormone levels (E2, testosterone, and others) in women with sexual dysfunction to a control group. This estimation took into account the prevalence of sexual dysfunction in approximately 50% of postpartum women, along with type 1 and type 2 error rates of 0.05 and 0.2, respectively^19^. The present research followed the STROBE guidelines for cohort studies, to ensure comprehensive reporting of study design, methodology, data collection, and analysis. By adhering to these standards, we aimed to enhance the transparency and rigor of our findings in epidemiological research^20^. This study applied strict inclusion and exclusion criteria to isolate biopsychosocial influences on postpartum FSIAD while minimizing bias and ensuring analytical accuracy. Inclusion criteria required participants to be literate, possess Iranian citizenship, carry a singleton pregnancy, have accessible electronic health records, and be free from chronic medical conditions or psychiatric disorders prior to pregnancy. Participants had to be in the third trimester without the onset of labor pains and have no history of FSIAD, as confirmed by the Female Sexual Function Index (FSFI). Exclusion criteria included undergoing instrumental vaginal delivery, experiencing severe neonatal illness or congenital malformations, the death of the infant or spouse, or divorce. Additionally, participants with postpartum depression (WHO-5 score < 50), any diagnosed illness, or use of medication within six months after delivery were excluded.

Participants completed questionnaires on demographic, socioeconomic, pregnancy history, and the questionnaire of Nonbiological Factors Influencing Sexual Desire in the third trimester of pregnancy (T1). The mothers’ body mass index (BMI) was calculated, and blood samples were collected to assess serum levels of adiponectin, E2, and testosterone.

The mothers were contacted and referred to the health centre for a prenatal visit at 30–42 days after delivery (T2). Mothers completed the FSFI, and their mode of childbirth was recorded. Blood samples were also taken.

Maternal weight and the time of first postnatal sexual intercourse were recorded when the subjects were referred to health centres to visit their 6-month-old baby (T3). Mothers completed the FSFI, and WHO-5 questionnaire. At this stage, blood samples were also taken.

Notably, throughout the cooperation, mothers’ questions about research or pregnancy-related problems, etc., were answered by phone. Counselling was provided in the case of a problem, and participants were referred if further counselling or treatment was needed.

### Measurement of hormone levels

All blood samples (3 cc) were collected from the antecubital vein by a trained clinician between 9:00 and 11:00 AM to control for diurnal variations in physiological markers^21,22^. Following collection, samples were centrifuged at 2000–3000 rpm for 10 min, and the separated serum was stored at − 80 °C until further analysis^21,23^. Zell Bio GmbH Human Adiponectin ELISA kits (Ulm, Germany) were used to measure adiponectin levels, while specific human ELISA kits were employed to quantify serum levels of estradiol (E2) and testosterone, following the manufacturers’ standardized protocols.

### Measurements of female sexual function index (FSFI)

A validated Persian version of the questionnaire on women’s sexual function was used. The reliability of the Persian version of the questionnaire was confirmed by a Cronbach’s alpha coefficient of 0.7^24^. This questionnaire consists of 19 questions; the first two questions are related to sexual desire, and the next four questions are related to arousal. Lower scores for the areas of desire (3.3) and sexual arousal (3.4) were used to diagnose the FSIAD^3^.

### The World Health Organization-Five Well-Being Index (WHO-5)

The WHO-5 questionnaire, known as a valid and reliable tool, was applied for psychological screening in Iranian pregnant mothers up to eight weeks postpartum (Cronbach’s alpha reliability coefficient was equal to 0.85 for the WHO-5 items)^25–27^. A review in 2015 identified the WHO-5 as a valid tool for screening for depression^26^. It consists of 5 questions about the participant’s feelings during the previous two weeks, with each item being scored on a 6-point Likert scale ranging from 0 to 5. The raw scores are transformed into a scale ranging from 0 to 100, where a score of 50 or less is indicative of a poor emotional state^25,28^. In our study, this scale was evaluated and found to have substantial reliability (0.81).

### Socioeconomic index

Three main factors related to socioeconomic status were used for constructing this index via Principal component analysis (PCA)^29^. These factors included economic indicators (occupation, spouse occupation, homeownership status, a separate bedroom for couples, number of bedrooms, indoor bathrooms, and cooking areas), asset indices (refrigerator, freezer, colour TV, washing machine, dishwasher, microwave, vacuum cleaner, personal car, landline, mobile phone, computer or laptop, and internet access), and social factors (education, spouse education, family members, and family supplementary health insurance)^27,29^.

### The questionnaire of nonbiological factors influencing sexual desire

It has 36 questions, of which 33 questions have been set in eight domains on a 5-point Likert scale. Each domain includes the following categories: marital relations, sexual attractiveness, spouse sexual function, socioeconomic status, motherhood, culture, privacy, and sexual experiences. The mean scores of the domains were the basis of comparison in the study. The correlation coefficient between the scores of the subjects in two shifts with an interval of two weeks was 0.87, and the Cronbach’s alpha coefficient of the questionnaire was 0.84^3^.

### Data analysis

MATLAB 2017 and SPSS-23 software were used for data analysis. Statistical tests, such as independent-sample t-tests, Mann‒Whitney U tests, and chi-square tests, were used to compare the FSIAD and control groups, with a P value < 0.05 considered significant. PCA was used to construct a socioeconomic index. The predictive model was designed by the C4.5 algorithm using input variables.

The input variables included type of childbirth, maternal age, maternal BMI (T1, T2, T3), waist circumference (T2, T3), serum adiponectin (T1, T2, T3), serum E_2_ (T1, T2, T3), serum testosterone (T1, T2, T3), breastfeeding status (T2, T3), sleep quality (T3), experiencing a stressful event in the past six months (T3), all domains of the questionnaire of nonbiological factors influencing sexual desire (T1) (including marital relationship, attractiveness, sexual skill of the wife, motherhood, culture, privacy, and sexual experiences), all domains of the FSFI (T3) (including lubrication, orgasm, satisfaction, and pain socioeconomic indices).The output variables, including FSIAD and normal sexual function, were coded as 0 and 1, respectively.

### Design of model

The decision tree algorithm was implemented and formed on the training data via the 70 − 30% method. The model was subsequently evaluated by training and testing the dataset^27^.

## Results

### Participants

**Fig. 1:**
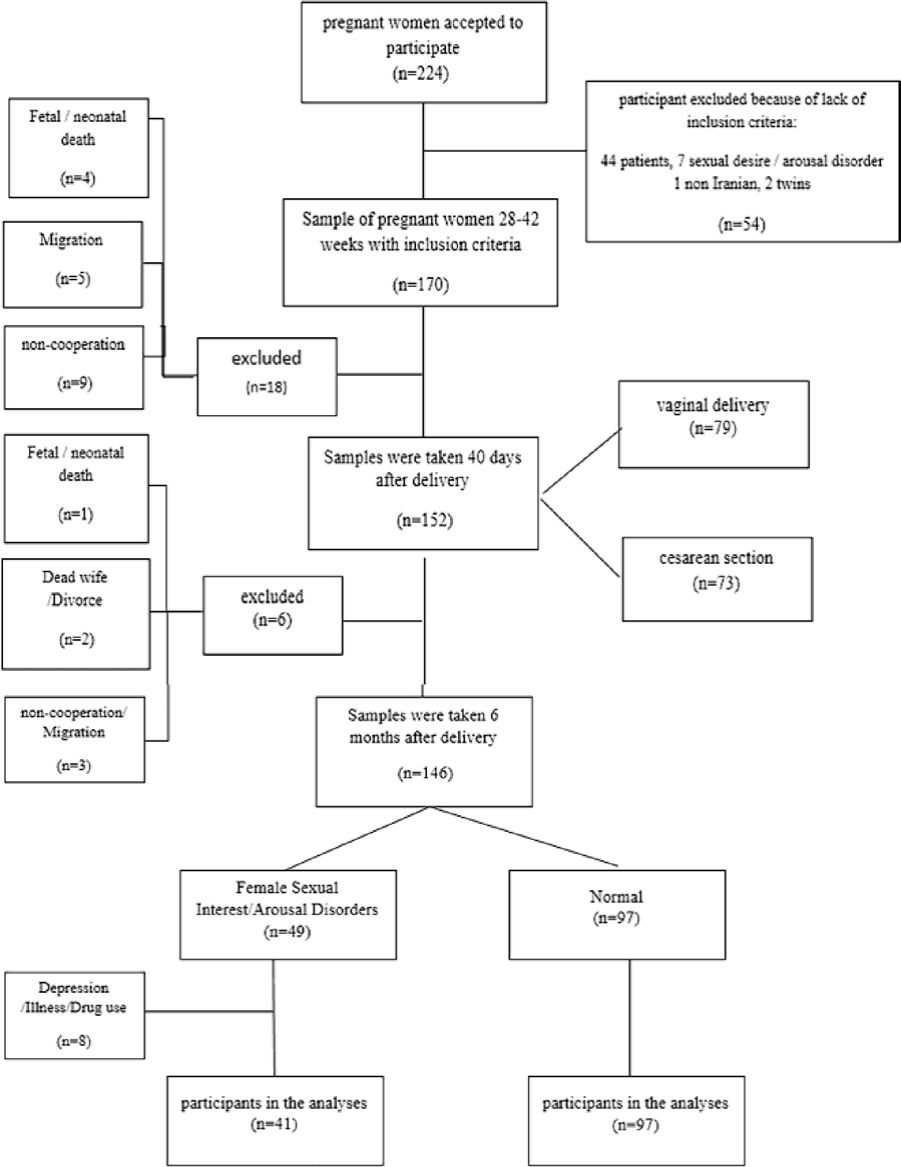
Follow-up of the mothers in the cohort.

A total of 170 eligible pregnant women were enrolled at baseline, of whom 138 completed all phases of the study and were included in the final analysis (Fig. 1). Mothers’ age was between 18 and 44 years, and the mean marriage duration was 6.7 ± 4.8 years. Almost all of the participants were households (84.1%) and had diplomas or upper levels of education (77%). In total, 42% of the subjects were nulliparous, and 27% had unwanted pregnancies. Attendance in childbirth preparation classes was low (28%), and only 14% of the training was about sexual problems in these classes. The mean gestational age at sampling time in the third trimester of pregnancy was approximately 31 weeks (range 28–39), and there was no significant difference in the mean gestational age between the normal and FSIAD groups. Table 1 shows the demographic and biopsychosocial characteristics of the two groups. In our study, waist circumference, levels of adiponectin (T2) and estrogen (T3), almost all dimensions of the questionnaire on nonbiological factors influencing sexual desire (including marital relationships, motherhood, attractiveness, sexual skill of the wife, and culture), and all domains of the FSFI were significantly different between the two groups (*p* < 0.05) (Tables 1 and 2). There were no other statistically significant differences in reporting.

**Table 1:** Comparison of biopsychosocial variables between the two groups.

	Variables	FSIAD *N* = 41	Normal *N* = 97	*P* value
Biological	Age (yr.) (M ± SD)	29.6 ± 5.5	28.9 ± 5.9	0.5
	Childbirth type (%)			
	VD	19 (46.3)	53 (54.6)	0.3
	CS	22 (53.7)	44 (45.4)	
	BMI (kg/m^2^) (M ± SD)			
	T1	29.8 ± 4.1	28.8 ± 4.1	0.1
	T2	27.7 ± 4.1	26.6 ± 3.8	0.1
	T3	26.9 ± 4.8	25.8 ± 4.4	0.2
	Waist size (cm) (M ± SD)			
	T2	98 ± 9.3	94 ± 10.2	0.03*
	T3	97.2 ± 11.3	92.9 ± 11.1	0.04*
	Adiponectin (µg/ml) (M ± SD)			
	T1	4.6 ± 1.4	4.4 ± 1.5	0.8
	T2	5.8 ± 2.1	4.8 ± 2	0.05*
	T3	4.8 ± 1.9	4.4 ± 2	0.1
	E2 (pg/ml) (M ± SD)			
	T1	3260 ± 1632.9	3351.4 ± 1356.5	0.9
	T2	38.7 ± 30	41.6 ± 57.6	0.3
	T3	34.9 ± 32.9	56.7 ± 62.5	0.00*
	Testosterone (ng/ml) (M ± SD)			
	T1	1.9 ± 1.4	2 ± 1.5	0.8
	T2	0.3 ± 0.2	0.2 ± 0.1	0.1
	T3	0.4 ± 0.3	0.3 ± 0.2	0.2
	Exclusive breastfeeding (%)			
	T2	35 (85.4)	85 (87.6)	0.3
	T3	34 (79.1)	84 (86.6)	0.5
Psychological	Sleep quality (%)			
	Very bad	-	1 (1)	0.7
	Bad	7 (17.1)	13 (13.4)	
	Good	26 (63.4)	57 (58.8)	
	Very good	8 (19.5)	26 (26.8)	
	Stressful event (%)			
	Yes	11 (26.8)	18 (18.6)	0.2
	No	30 (73.2)	79 (81.4)	
	Marital relationship (M ± SD)	2.4 ± 0.3	2.5 ± 0.3	0.04*
	Motherhood (M ± SD)	2.8 ± 0.5	2.6 ± 0.5	0.05*
	Attractiveness (M ± SD)	3.7 ± 0.6	3.9 ± 0.6	0.03*
	Sexual experience (M ± SD)	1.7 ± 0.3	1.7 ± 0.3	0.4
	Sexual skill of wife (M ± SD)	3.7 ± 0.7	3.9 ± 0.7	0.02*
Social	Socioeconomic status index (%)			
	Low	12 (29.3)	23 (23.7)	0.5
	Medium	18 (43.9)	53 (54.6)	
	High	11 (26.8)	21 (21.6)	
	Culture (M ± SD)	3 ± 0.6	3.3 ± 0.8	0.01*
	Privacy (M ± SD)	3.1 ± 1.3	3.1 ± 1.2	0.8

VD: vaginal delivery, CS: cesarean section

**Table 2:** Comparison of FSFI scores between the two groups.

FSFI	FSIAD *N* = 41	Normal *N* = 97	*P* value
Lubrication disorder	14 (34.1%)	3 (3.1%)	< 0.001*
Orgasmic disorder	16 (39%)	1 (1%)	< 0.001*
Dissatisfaction	10 (24.4%)	3 (3.1%)	< 0.001*
Sexual pain disorder	12 (29.3%)	3 (3.1%)	< 0.001*

### Building the model

The sensitivity, specificity, and accuracy of the model were 97.17, 95.50 and 96.98%, respectively, in the training phase, and they were 95.92, 86.08 and 93.7%, respectively, in the evaluation phase. Orgasm (T3), adiponectin (T1), waist circumference (T2, T3), estrogen level (T3), and pain (T3) were the most significant factors for predicting interest/arousal disorders at six months after childbirth.

**Fig. 2:**
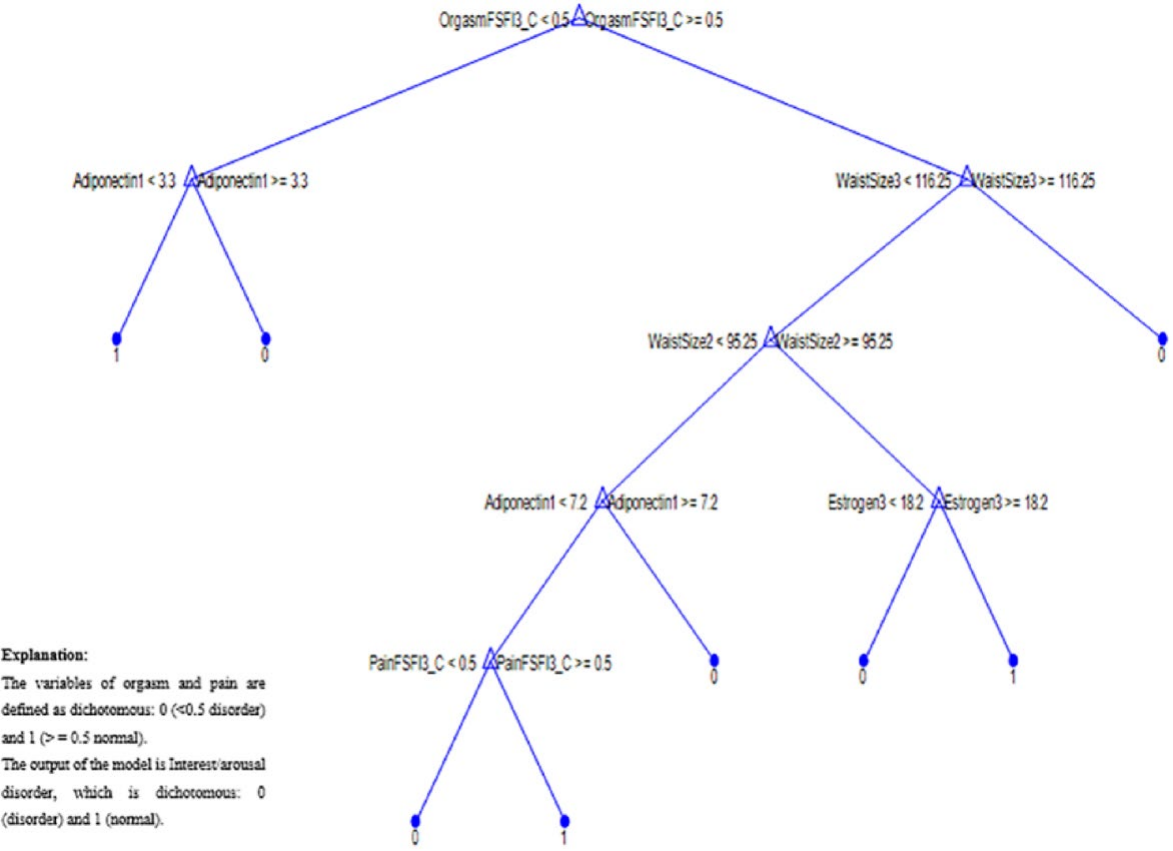
The proposed tree structure.

Figure 2 shows the tree structure and presents the set of rules created by the C5 model. Table 3 presents rules resulting from the model that consist of several logical implications (if-then rule).

**Table 3:** Rules and their explanations.

Sr. No	Rules	Confidence
1	(Orgasm 3 = disorder and Adiponectin 1 < 3.3) = > Normal interest/arousal	100%
If there is an orgasmic disorder within six months after delivery and serum level of adiponectin in the third trimester of pregnancy is less than 3.3, then the probability of normal sexual interest/arousal is equal to 100% within six months after delivery		
2	(Orgasm 3 = disorder and Adiponectin 1 ≥ 3.3) = > Interest/arousal disorder	100%
If there is an orgasmic disorder within six months after delivery and the serum level of adiponectin in the third trimester of pregnancy is more than 3.3, then the probability of interest/arousal disorder is equal to 100% within six months after delivery		
3	(Orgasm 3 = normal and Waist circumference 3 ≥ 116.25) = > Interest/arousal disorder	100%
If orgasm is normal within six months after delivery and waist circumference is greater than 116.25, then the probability of interest/arousal disorder is equal to 100% within six months after delivery		
4	(Orgasm 3 = normal and Waist circumference 3 < 116.25 and Waist circumference 2 ≥ 95.25 and Estrogen3 ≥ 18.2) = > Normal interest/arousal	81.63%
If orgasm is normal within six months after delivery, waist circumference is less than 116.25, waist circumference within 40 days postpartum is greater than 95.25 and serum estrogen level within six months postpartum is greater than 18.2, then the probability of normal interest/arousal is equal to 81.63% within six months after delivery		
5	(Orgasm 3 = normal and Waist circumference 3 < 116.25 and Waist circumference 2 ≥ 95.25 and Estrogen 3 < 18.2) = > Interest/arousal disorder	100%
If orgasm is normal within six months after delivery, waist circumference within six months postpartum is less than 116.25, waist circumference within 40 days postpartum is greater than 95.25, and serum estrogen level within six months postpartum is less than 18.2, then the probability of interest/arousal disorder is equal to 100% within six months after delivery		
6	(Orgasm 3 = normal and Waist circumference 3 < 116.25 and Waist circumference 2 < 95.25 and Adiponectin 1 ≥ 7.2) = > Interest/arousal disorder	
If orgasm is normal within six months after delivery, waist circumference within six months after delivery is less than 116.25, waist circumference within 40 days after delivery is less than 95.25, and serum adiponectin level in the third trimester of pregnancy is greater than 7.2, then the probability of interest/arousal disorder is equal to 66.67% within six months after delivery		
7	(Orgasm 3 = normal and Waist circumference 3 < 116.25 and Waist circumference 2 < 95.25 and Adiponectin 1 < 7.2 and Pain 3 = disorder) = > interest/arousal disorder	100%
If orgasm is normal within six months after delivery, waist circumference within six months after delivery is less than 116.25, waist circumference within 40 days after delivery is less than 95.25, serum adiponectin level in the third trimester of pregnancy is less than 7.2, and pain disorder exists within six months after delivery, then the probability of interest/arousal disorder is equal to 100% within six months after delivery		
8	(Orgasm 3 = normal and Waist circumference 3 < 116.25 and Waist circumference 2 < 95.25 and Adiponectin 1 < 7.2 and Pain 3 = normal) = > normal interest/arousal	94.83%
If orgasm is normal within six months after delivery, waist circumference within six months after delivery is less than 116.25, waist circumference within 40 days after delivery is less than 95.25, serum adiponectin level in the third trimester of pregnancy is less than 7.2, and pain level is normal then the probability of interest/ normal arousal is equal to 94.83%within six months after delivery		

## Discussion

This cohort study was designed in accordance with standard methodological steps of cohort studies. A group of healthy pregnant women in their third trimester—with no prior diagnosis of FSIAD or mental illness—was recruited. These women were followed at two time points—40 days and six months postpartum—to monitor changes in relevant variables and assess the development of postpartum FSIAD. At six months, the incidence of FSIAD was evaluated, and biopsychosocial predictors were compared between participants who developed the disorder and those who did not. A total of 29.7% of the women developed FSIAD by six months after delivery, a prevalence lower than that reported in earlier studies, which ranged from 52.3–81.2%^2,29^. This difference may be attributable to stricter inclusion criteria, including the exclusion of participants with pre-existing sexual dysfunction or depression, as well as the use of the DSM-5 diagnostic framework, which consolidates desire and arousal disorders into a unified category^5^. Using the C4.5 decision tree algorithm, our study achieved a high predictive accuracy (93.7%) in identifying FSIAD cases. This method allowed us to identify a set of key predictors, namely elevated adiponectin levels, reduced estradiol levels, increased waist circumference, orgasmic dysfunction, and the presence of pain. These findings suggest that specific biological and functional factors may serve as more reliable indicators of FSIAD than commonly presumed demographic or psychosocial variables. Among the most notable findings was the strong association between increased adiponectin levels and FSIAD. Adiponectin, an adipokine secreted primarily by adipose tissue, has known roles in metabolic regulation, inflammation, and neuroendocrine signalling. It is also expressed in reproductive tissues and the central nervous system^10,30^. The neuroprotective and antidepressant-like properties of adiponectin may be mediated by its receptors in the CNS^31^and disruptions in adipokine secretion have been linked to psychiatric disorders, including postpartum depression^12^. Prior studies have found elevated adiponectin in women with postpartum depression and reduced marital satisfaction^32,33^both of which are known correlates of sexual dysfunction. These findings support the hypothesis that increased adiponectin may reflect an underlying psychoneuroendocrine imbalance contributing to FSIAD. Moreover, adiponectin is closely linked to a range of reproductive hormones, including estrogens, androgens, sex hormone-binding globulin (SHBG), insulin-like growth factor-1 (IGF-1), and prolactin^8^all of which influence female sexual function. Notably, menopause, a condition characterized by hypoestrogenism, shares symptomatic similarities with FSIAD. Studies have reported a significant positive correlation between adiponectin and follicle-stimulating hormone (FSH), and a negative correlation with estrogen, independent of body mass index (BMI) and age, in postmenopausal women^34^. Hormonal alterations during menopause, particularly the decline in estrogen levels, are associated with an increase in total body adiposity, especially visceral fat accumulation. This redistribution of fat is often accompanied by upregulation of adiponectin, peroxisome proliferator-activated receptor gamma (PPARγ), and fatty acid transporters in gluteal adipose tissue. Such metabolic adaptations may represent a compensatory physiological mechanism aimed at maintaining systemic insulin sensitivity in estrogen-deficient postmenopausal women. Taken together, our findings point to adiponectin as a potential biomarker involved in the neuroendocrine regulation of postpartum sexual function. In our study, the probability of FSIAD increased with decreasing levels of estradiol (E2). Estrogen plays a crucial role in female sexual function and libido, and a minimum serum concentration of approximately 50 pg/mL is generally considered necessary to support changes in genital sensation, vaginal lubrication, and enhancement of sexual responsiveness. Estrogen exerts its influence through multiple neuroendocrine pathways, including modulation of the cholinergic system, neuropeptide systems (such as melanocortin, opioid, and oxytocin), the gamma-aminobutyric acid (GABA) neurotransmitter, and the synthesis and transport of monoamines such as dopamine, noradrenaline, and serotonin^7^. Moreover, estrogen promotes genital vasodilation by stimulating the release of nitric oxide (NO) from endothelial cells, leading to the activation of soluble guanylate cyclase and the formation of cyclic guanosine monophosphate (cGMP), a key mediator of the local excitatory responses observed during sexual arousal^35^. These mechanisms support estrogen’s central role in maintaining healthy sexual desire and arousal, and may account for the inverse relationship between E2 levels and FSIAD identified in our model. In addition, our findings indicated a positive association between increasing waist circumference and the likelihood of developing FSIAD. Prior studies have shown that a slimmer waist circumference is associated with higher levels of sexual satisfaction, more frequent vaginal intercourse, and improved erectile function in both partners, even after controlling for age. Furthermore, women with central (abdominal) obesity are more likely to experience impairments in vaginal orgasm, as opposed to orgasm achieved through other forms of stimulation. Several potential mechanisms have been proposed to explain these associations: neurohormonal alterations resulting from increased adiposity may directly diminish female sexual desire, while abdominal obesity may also lower perceived sexual attractiveness, thereby reducing a partner’s libido via evolutionarily rooted preferences. Given that vaginal orgasm is more dependent on mutual physical health and fitness, these interconnected biological and psychosocial factors may collectively mediate the adverse impact of increased waist circumference on sexual interest and arousal^36,37^. Of particular note in our study was the strong association between orgasmic dysfunction and FSIAD, echoing findings from previous research which reported diminished sexual desire following impairments in orgasmic response^38^. This suggests a potential causal or reinforcing relationship between these two dimensions of female sexual function, warranting further investigation. Our study identified pain disorders as a significant predictor of FSIAD. This finding aligns with the broader literature emphasizing the bidirectional relationship between pain and sexual dysfunction. One potential biological mechanism underlying this association involves estrogen deficiency. As noted earlier, participants in the FSIAD group exhibited lower levels of estradiol. Estrogen plays a key role in maintaining vaginal tissue integrity and moisture, and its deficiency may result in vaginal atrophy and dryness, leading to dyspareunia (painful intercourse)^39^. Pain during sexual activity can directly inhibit sexual desire by creating anticipatory anxiety and avoidance behaviors, thereby contributing to the onset or persistence of FSIAD. Despite the multifactorial nature of female sexual dysfunction reported in previous studies, our model identified pain and a subset of biological factors (e.g., estrogen, waist circumference, adiponectin, and orgasm function) as more critical in predicting FSIAD six months postpartum. While factors such as maternal age, mode of delivery, BMI, testosterone levels, breastfeeding, sleep quality, recent stress, marital relationship quality, feelings of motherhood, self-perceived attractiveness, previous sexual experience, sexual skills, cultural and social norms, and other FSFI domains like lubrication and satisfaction were not significant predictors in our model, there may be contexts where some of these variables are statistically associated with postpartum FSIAD. Our model employs a non-linear decision tree algorithm (C4.5) that selects predictors based on their contribution to classification accuracy using information gain. It is important to note that statistical significance in isolation does not guarantee predictive power in a multivariate, non-linear model. Some variables may show group differences but offer limited incremental value when added to a model that already includes stronger or more informative predictors. Additionally, although more complex models such as ensemble classifiers could potentially incorporate a broader range of variables, they often lack interpretability and transparency. In contrast, we chose a decision tree algorithm because it provides a clinically interpretable structure, which is essential for real-world medical decision-making and early intervention planning.

### Limitations of the study

One of the strengths of this study is its longitudinal design, which allows for an assessment of women during the third trimester of pregnancy. Additionally, hormonal assessments of the mothers were conducted at three different time intervals, providing depth to the findings. In the initial phase of the study, we aimed to include women who were medically and psychiatrically healthy. However, there were some limitations, including a follow-up rate loss of approximately 14%. Additionally, there is a possibility of recall bias, as women were asked to report their sexual function before pregnancy during the initial screening. It is also worth noting that levels of LH and FSH were not measured in this study.

## Conclusion

This study is the first to apply a decision tree model using biopsychosocial factors—particularly serum adiponectin—to predict Female Sexual Interest/Arousal Disorder (FSIAD) in the postpartum period. With a prevalence rate of 29.7% at six months postpartum, the C4.5 algorithm demonstrated high predictive accuracy by identifying key predictors, elevated serum adiponectin, reduced estrogen levels, increased waist circumference, orgasm and pain disorder. The decision tree model developed in this study offers a practical and interpretable tool for the early identification of women at risk for FSIAD in the postpartum period. By leveraging simple and measurable factors, the model can be integrated into routine postpartum care to support early clinical decision-making. Its application may facilitate timely, individualized interventions and targeted counselling before the disorder becomes chronic or begins to adversely impact quality of life and intimate relationships. Furthermore, the model’s clarity and accessibility make it especially valuable in low-resource settings where specialized psychological assessments are not readily available. However, larger and more diverse cohort studies are needed to validate and adapt this model across different populations and healthcare settings.

## Data Availability

The datasets used and/or analyzed during the current study are available from the corresponding author on reasonable request.

## Abbreviations

FSIAD: Female sexual interest/arousal disorders
FSFI: Female sexual function index
WHO-5: The World Health Organization- Five Well-Being Index
DSM5: Diagnostic and Statistical Manual of Mental Disorders
HSDD: Hypoactive sexual desire disorder
E2: Estradiol E2
T1: Their third trimester of pregnancy
T2: 30–42 days after delivery
T3: 6-month-old baby
BMI: Body mass index
ELISA: Enzyme-linked immunosorbent assay
PCA: Principal component analysis
VD: Vaginal delivery
CS: Cesarean section
CNS: Central nervous system
SHBG: Sex hormone binding globulin
IGF-1: Insulin-like growth factor-1
FSH: Follicle-stimulating hormone
LH: Luteinizing hormone
PPARγ: Peroxisome proliferator-activated receptorγ
GABA: Gamma aminobutyric acid
NO: Nitric oxide
cGMP: Cyclic guanosine monophosphate
